# The role of Europe in Global Psychiatry

**DOI:** 10.1192/j.eurpsy.2024.28

**Published:** 2024-08-27

**Authors:** P. Falkai

**Affiliations:** Psychiatry, University of Munich, Munich, Germany

## Abstract

**Abstract:**

The burden of mental illness in Europe: High individual and societal burden, but only 2% spent on mental health. The tradition of European Psychiatry needs to be strengthened in care, research and teaching.Within the long-term Strategic Mental Health Plan of the EPA the improvement of clinical care research, the “mapping excellence” and “developing core treatment guidelines” require further action. Researching the influence of environmental stressors on the development and maintenance of mental illness and fostering stepped care approaches to improve resilience are none the less important. Furthermore, to improve the reliability and especially validity of diagnoses of mental disorders by introducing (bio)markers and defining dimensions of mental illnesses using big data and predictive sciences are just as important as an enforced research on reducing stigma and discrimination of mental disorders.

**Disclosure of Interest:**

None Declared

